# Identifying individualized prognostic signature and unraveling the molecular mechanism of recurrence in early-onset colorectal cancer

**DOI:** 10.1186/s40001-023-01491-y

**Published:** 2023-11-20

**Authors:** Jia Yang, Yuting Zhao, Rongqiang Yuan, Yongtong Wang, Shiyi Wang, Zhiqiang Chang, Wenyuan Zhao

**Affiliations:** Department of Systems Biology, College of Bioinformatics Science and Technology, Harbin Medical University, Harbin, 150086 China

**Keywords:** Early-onset colorectal cancer, Prognostic signature, Relative expression ordering, Recurrence-associated genes, Immune microenvironment

## Abstract

**Background:**

The incidence and mortality of early-onset colorectal cancer (EOCRC; < 50 years old) is increasing worldwide, with a high recurrence rate. The inherent heterogeneity of EOCRC makes its treatment challenging. Hence, to further understand the biology and reveal the molecular mechanisms of EOCRC, a recurrence risk signature is needed to guide clinical management.

**Methods:**

Based on the relative expression orderings (REOs) of genes in each sample, a prognostic signature was developed and validated utilizing multiple independent datasets. The underlying molecular mechanisms between distinct prognostic groups were explored via integrative analysis of multi-omics data.

**Results:**

The prognostic signature consisting of 6 gene pairs (6-GPS) could predict the recurrence risk for EOCRC at the individual level. High-risk EOCRC classified by 6-GPS showed a poor prognosis but a good response to adjuvant chemotherapy. Moreover, high-risk EOCRC was characterized by epithelial-mesenchymal transition (EMT) and enriched angiogenesis, and had higher mutation burden, immune cell infiltration, and *PD-1*/*PD-L1* expression. Furthermore, we identified four genes associated with relapse-free survival in EOCRC, including *SERPINE1*, *PECAM1*, *CDH1*, and *ANXA1*. They were consistently differentially expressed at the transcriptome and proteome levels between high-risk and low-risk EOCRCs. They were also involved in regulating cancer progression and immune microenvironment in EOCRC. Notably, the expression of *SERPINE1* and *ANXA1* positively correlated with M2-like macrophage infiltration.

**Conclusion:**

Our results indicate that 6-GPS can robustly predict the recurrence risk of EOCRC, and that *SERPINE1*, *PECAM1*, *CDH1*, and *ANXA1* may serve as potential therapeutic targets. This study provides valuable information for the precision treatment of EOCRC.

**Supplementary Information:**

The online version contains supplementary material available at 10.1186/s40001-023-01491-y.

## Background

Globally, colorectal cancer (CRC) is the third most commonly diagnosed cancer and is the second cause of cancer-related death. Over the past decades, the incidence and mortality of sporadic CRC have declined globally due to improved screening and treatment methods [1, 2]. However, epidemiological data shows the morbidity of early-onset CRC (EOCRC) is increasing worldwide. Currently, nearly one-fifth of new CRC cases occur in individuals aged 50 years or younger [3].

Many studies have shown differences in the clinicopathological features of EOCRC and late-onset CRC (LOCRC; $\ge$ 50 years old). Compared with LOCRC, EOCRC mainly occurs in the rectum and distal colon and is more likely to be diagnosed in advanced stages (stage III-IV). EOCRC also has more advanced pathological features such as poor differentiation, perineural infiltration, and signet ring cell formation [4–6]. In addition, many studies have reported that patients with EOCRC tend to have worse relapse-free survival. Early age of onset is an independent unfavorable predictor [7–9]. There are several studies have also reported heterogeneity in the molecular characteristics of EOCRC [6, 10–12]. For example, the distribution of consensus molecular subtypes (CMS) of CRC may be related to age of onset, compared to LOCRC patients aged 50–69 years (11% CMS1), EOCRC patients under 50 years of age had a higher prevalence of CMS1 (22–23% CMS1). Whereas CMS1 was the most prevalent subtype in patients younger than 40 years, CMS3 and CMS4 were infrequent. Patients aged 18 to 29 years had fewer APC mutations and a higher prevalence of signet ring histology compared with other patients younger than 50 years [13]. Therefore, heterogeneous subgroups may exist in EOCRC.

As the number of cases with EOCRC continues to increase, there is an urgent need to optimize cancer treatment strategies. The high recurrence rate of EOCRC is an important concern, but the mechanisms of recurrence are currently unknown. Therefore, it is necessary to develop a novel and effective biomarker to stratify the recurrence risk of EOCRC, thereby enabling more personalized management. Currently, there is a prognostic nomogram model for patients with early-onset stage I–II colon cancer [14]. Similarly, there is a risk prediction model combining genetic and environmental risk scores for patients with EOCRC [15], but they are influenced by batch effects of cohorts. In addition to batch effects, these models are not appropriate for the individual patient.

In our previous studies, we established several personalized signatures for individualized testing based on relative expression orderings (REOs) of genes in a sample that are highly robust to the experimental batch effect [16–18]. In addition, REO-based signatures can be applied to the individual patient [19, 20].

In this study, based on the REOs of genes in each sample, we developed an individualized and qualitative [21] transcriptional signature for predicting the recurrence risk of patients with EOCRC. We further explored the impact of clinical features, multi-omics molecular characteristics, and immune microenvironment on the recurrence of EOCRC. This prognostic signature may help identify high-risk EOCRC patients and assist clinicians in making better decisions for treating patients.

## Methods

### CRC patient cohort

In this study, gene expression profiles of CRC and corresponding clinical information were downloaded from the Gene Expression Omnibus database (GEO, http://www.ncbi.nlm.nih.gov/geo) and cBioportal (https://www.cbioportal.org/). We defined patients younger than 50 years as early-onset CRC and patients older than 60 years as late-onset CRC. CRC samples from The Cancer Genome Atlas (TCGA) and GSE17538, which contain complete survival information, were utilized as training cohorts to establish a recurrence risk signature. The signature was validated using GSE39582 and GSE14333. In addition, using GSE72970 and GSE104645, we analyzed the response of patients with EOCRC to adjuvant chemotherapy (ACT). Somatic mutation, copy number aberration (CNA), and proteomics reverse phase protein array data for EOCRC were obtained from cBioportal. Table 1 provides details of these datasets.

**Table 1:** Data used in this study

Data	Data type	EOCRC samples	LOCRC samples	Source
GSE17538	mRNA	26	128	http://www.ncbi.nlm.nih.gov/geo
GSE39582	mRNA	65	–	http://www.ncbi.nlm.nih.gov/geo
GSE14333	mRNA	24	–	http://www.ncbi.nlm.nih.gov/geo
GSE72970	ACT response	15	–	http://www.ncbi.nlm.nih.gov/geo
GSE104645	ACT response	28	–	http://www.ncbi.nlm.nih.gov/geo
TCGA	mRNA	74	401	https://www.cbioportal.org/
TCGA	Somatic mutation	69	–	https://www.cbioportal.org/
TCGA	DNA copy number	74	–	https://www.cbioportal.org/
TCGA	Protein	56	–	https://www.cbioportal.org/

### Developing the REO-based recurrence risk signature in EOCRC

**Fig. 1:**
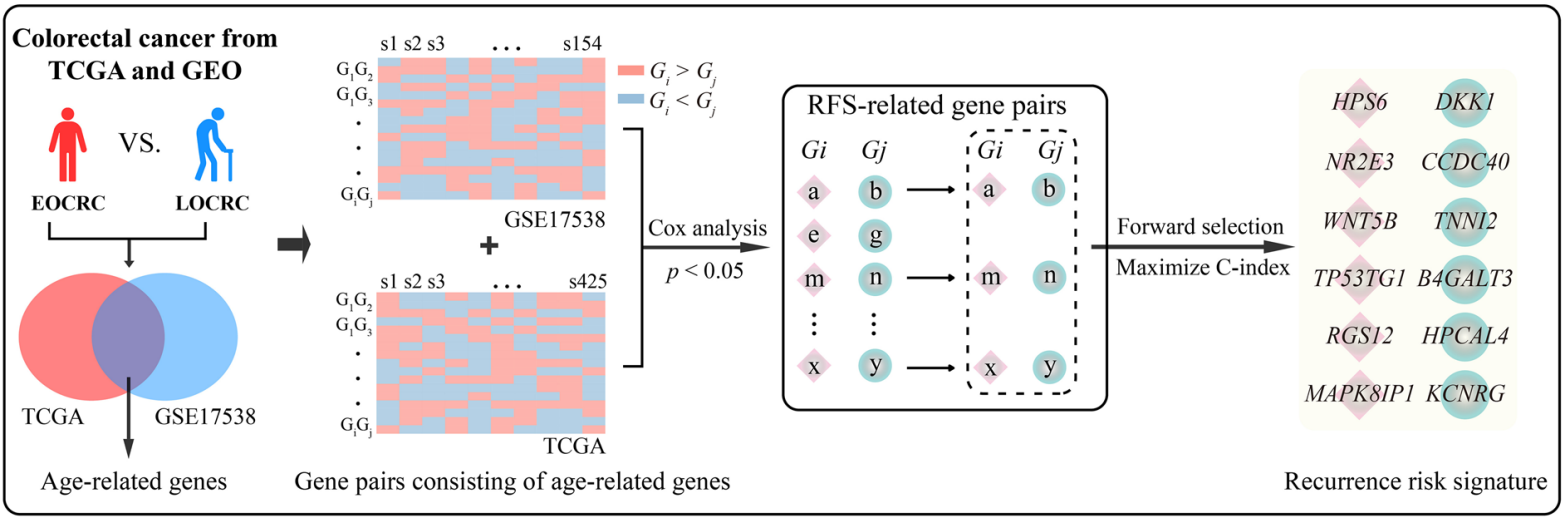
The flowchart of developing REO-based recurrence risk signature

The process of developing REO-based recurrence risk signature is described in Fig. 1.

First, in the training sets TCGA and GSE17538, differentially expressed genes (DEGs) between EOCRC and LOCRC were identified using the Wilcoxon rank-sum test (*p* < 0.05), respectively. The overlapping genes between two lists of DEGs were defined as age-related genes. Pairwise comparisons were performed for the expression level of age-related genes in each sample. For gene pairs composed of age-related genes, *G*_*i*_ and *G*_*j*_ represented the expression values of gene *i* and gene *j*, respectively. For each gene pair (*G*_*i*_, *G*_*j*_), with only two possible REO patterns (*G*_*i*_ > *G*_*j*_ or *G*_*i*_ < *G*_*j*_).

Second, for a sample, the label of the sample is specified as 1 if a gene pair with REO of *G*_*i*_ > *G*_*j*_, otherwise 0; then, based on the univariate Cox proportional hazards model, gene pairs were detected with specific REO significantly correlated with relapse-free survival (RFS) in surgery-only patients from the TCGA and GSE17538 (*p* < 0.05). After that, we identified a panel of consistent prognosis-related gene pairs overlapping in the two datasets. A gene may be involved in multiple prognosis-related gene pairs, thus for gene pairs sharing the same gene, we only kept the gene pair with the most significant *p*-value to avoid redundancy.

Third, in surgery-only EOCRC from the TCGA, a forward-stepwise selection algorithm was applied to find the optimal subset of gene pairs that led to the highest concordance index (C-index) [22] among the candidate prognosis-related gene pairs following the half-voting rule. Starting with the gene pair with the largest C-index as the seed signature, a candidate gene pair were added to the signature one at a time until the addition of any one gene pair failed to increase the C-index. The optimal subset of gene pairs was defined as the recurrence risk signature in EOCRC.

Based on the REO pattern of gene pairs (*G*_*i*_ > *G*_*j*_ or *G*_*i*_ < *G*_*j*_), a sample was identified as high-risk if more than half of the REOs of gene pairs in the recurrence risk signature voted for high-risk; otherwise, this sample was assigned to the low-risk group.

### Consensus molecular subtypes

A molecular subtype was assigned to each CRC sample based on the gene expression spectrum of the TCGA dataset using the random forest classifyCMS function in the “CMSclassifier” R package [23] (https://github.com/Sage-Bionetworks/CMSclassifier).

### Multi-omics analysis

DEGs and differentially expressed proteins between high-risk and low-risk groups were identified using the limma algorithm [24]. False discovery rate (FDR) < 0.05 was considered as the threshold for DEGs. The ComplexHeatmap [25] was used to show the top 20 differentially mutant genes. Fisher’s exact test was performed to determine significantly different mutant genes and significantly higher frequent CNA between the high-risk and low-risk groups. Tumor mutational burden (TMB) was defined as mutations per million bases and calculated by the “maftools” R package. CNA fraction and aneuploidy scores were derived from Thorsson et al. [26]. *P*-value < 0.05 was considered statistically significant.

### Functional enrichment analysis

The single-sample gene set enrichment analysis (ssGSEA) was performed to calculate the enrichment scores of hallmark gene sets for the high-risk and low-risk groups. The Wilcoxon rank-sum test was utilized to identify significantly differential pathways, considering *p*-value < 0.05. GSEA analysis was performed on the high-risk and low-risk groups. Genes were ranked according to the fold change in their expression in the samples of the two groups. Then, we investigated whether hallmark gene sets were significantly enriched at the top or bottom of the ranked list, with *p*-value < 0.05 considered significant. Kyoto Encyclopedia of Genes and Genomes (KEGG) enrichment analysis of DEGs and differentially expressed proteins was performed using the “clusterProfiler” R package. Hallmark gene sets were obtained from the Molecular Signatures Database (MSigDB). *P*-value < 0.05 was considered statistically significant.

### Protein–protein interaction network analysis

A PPI network consisting of DEGs was constructed using the STRING v11.5 [27] (https://string-db.org/) with a confidence score > 0.4. The Cytoscape software (https://cytoscape.org/) was used to visualize the network, and MCODE (Molecular complex detection) plugin was applied to cluster the PPI network based on topology and find densely connected regions.

### Immune landscape analysis

The immune score and stromal score were calculated using “estimate” R package [28] based on expression profile data. TIMER [29], CIBERSORT [30], xCell [31], quanTIseq [32], and MCP-counter [33] were applied to estimate the proportion of infiltrating immune cells on TIMER 2.0 (http://timer.cistrome.org/). T-cell receptor (TCR) diversity measured by Shannon entropy that can predict the response of patients to immunotherapy and leukocyte fraction were obtained from the study by Thorsson et al. [26]. Cytolytic activity was obtained from the study by Rooney et al. [34]. The biomarkers of adaptive immune cells, innate immune cells, inflammation promoting, and human leukocyte antigen (HLA) were selected from a previous study [35], and scores were calculated using ssGSEA. Wilcoxon rank-sum test was used to compare the immune scores, stromal scores, proportion of immune cell infiltration proportion, and immune-related signature scores between high-risk and low-risk samples. Pearson’s correlation test was used to calculate correlation coefficients (*r*) and *p*-value. *P*-value < 0.05 was considered statistically significant.

### Survival analysis

We drew the survival curve using the Kaplan–Meier method and compared survival differences using the log-rank test. We calculated C-index, hazard ratio (HR), and 95% confidence interval (CI) using the univariate Cox proportional hazards model. Multivariate Cox regression analysis was used to verify the independence of the prognostic signature. *P*-value < 0.05 was considered statistically significant.

### Statistical analysis

All statistical analyses in this study were performed using R software version 4.2.1 (http://www.r-project.org/).

## Results

### Age affects the survival of patients with CRC

Consistent with previous studies, we found a higher proportion of stage III and IV patients with EOCRC compared to patients with LOCRC in TCGA (*p* = 0.024; Fisher’s exact test; Additional file 1: Table S1).

Furthermore, we compared survival between patients with EOCRC and LOCRC. For four datasets (TCGA, GSE39582, GSE17538, and GSE14333) with RFS/disease-free survival (DFS) information, EOCRC showed poorer RFS/DFS than LOCRC in two of these datasets (Additional file 1: Fig. S1C, D). TCGA, GSE39582, and GSE17538 datasets also contained overall survival (OS) information, conversely, EOCRC showed better OS than LOCRC in two of these datasets (Additional file 1: Fig. S1E, F). These results suggest that patients with EOCRC have a better OS but a higher recurrence risk compared to patients with LOCRC, indicating that age is associated with the recurrence of CRC. Hence, we constructed a signature to predict the risk of EOCRC recurrence based on age-related DEGs between EOCRC and LOCRC patients.

### The REO-based recurrence risk signature for EOCRC

Figure 1 depicts the flowchart of developing REO-based recurrence risk signature. First, we identified 1994 and 2869 DEGs between EOCRC and LOCRC samples from TCGA and GSE17538, respectively (*p* < 0.05; Wilcoxon rank-sum test). We screened 247 overlaps between the two sets of DEGs to define as age-related genes. Next, using surgery-only EOCRC and LOCRC in TCGA (*n* = 452) and GSE17538 (*n* = 154), we identified 2499 and 1592 gene pairs consisting of age-related genes, the REO patterns of which were significantly associated with RFS of patients (*p* < 0.05; univariate Cox proportional hazards model). After that, we found 91 gene pairs with consistent REO patterns between the above two sets of gene pairs. Twenty-two gene pairs were retained following de-redundancy of the gene pairs. Then, using a forward-stepwise selection algorithm, we identified 6 gene pairs with the highest C-index of 0.828 in the surgery-only EOCRC patients from the TCGA (*n* = 66) (see “Materials and methods”). Finally, the 6 gene pairs (6-GPS) were defined as the recurrence risk signature for EOCRC (Additional file 1: Table S2). A patient was classified into the high-risk group if more than 3 of the 6-GPS were in favor of high-risk; otherwise, the patient was classified into the low-risk group.

**Fig. 2:**
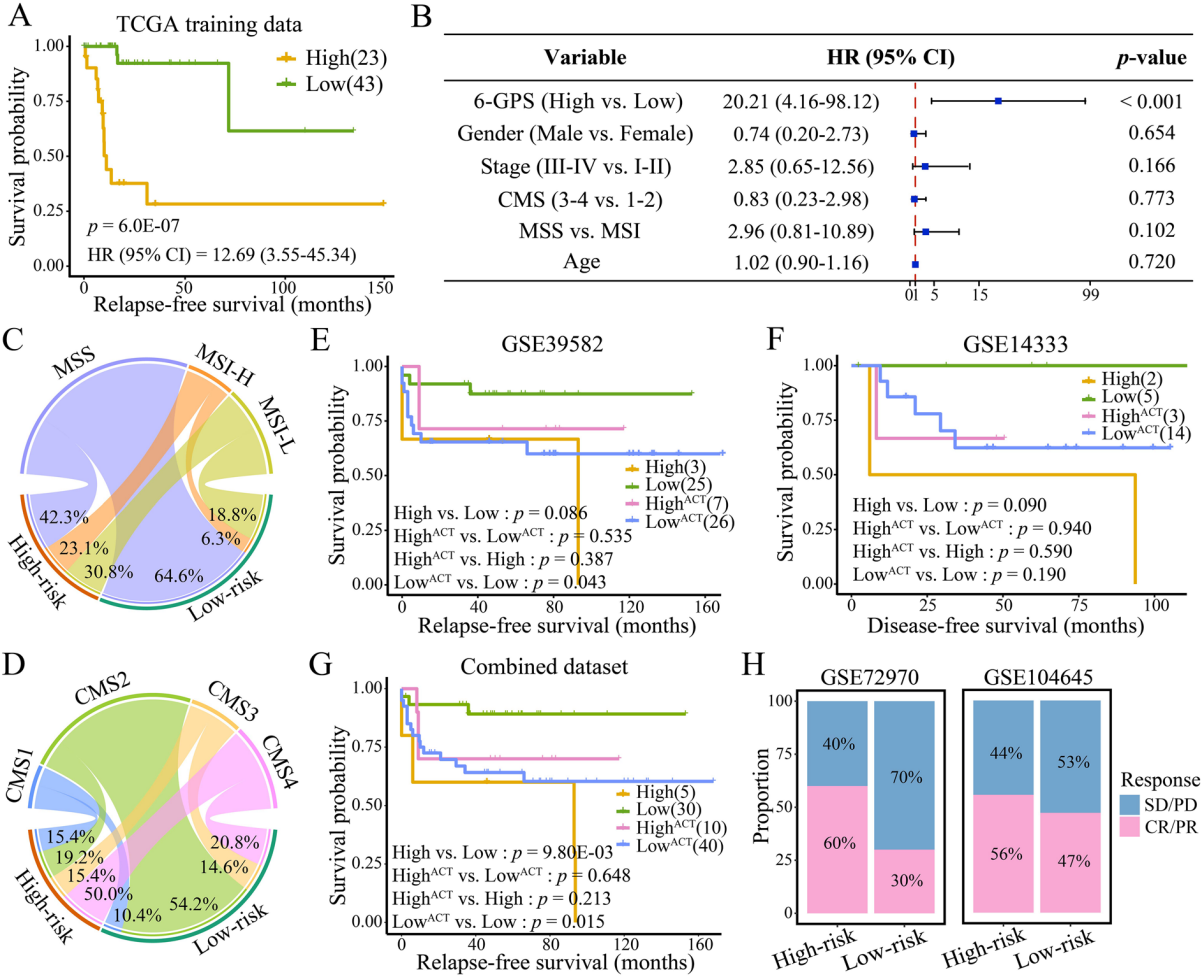
The performance of 6-GPS. **A** Kaplan–Meier curves depicting the survival difference between high-risk and low-risk EOCRCs classified by 6-GPS in TCGA. High, high-risk samples with surgery-only; Low, low-risk samples with surgery-only. **B** Multivariate Cox regression analysis in TCGA. **C**, **D** Circos plots showing the proportion of molecular subtypes in high-risk and low-risk EOCRCs. Kaplan–Meier curves depicting the survival difference between high-risk and low-risk EOCRCs in GSE39582 (**E**), GSE14333 (**F**), and combined dataset (**G**). High^ACT^, high-risk samples receiving ACT; Low^ACT^, low-risk samples receiving ACT. **H** Histograms showing the difference in response to ACT between high-risk and low-risk samples in GSE72970 and GSE104645. SD/PD: stable/progressive disease; CR/PR: complete/partial response

In the TCGA training cohort with 66 surgery-only EOCRC samples, 23 were classified into the high-risk group, and 43 were classified into the low-risk group by 6-GPS. Survival analysis showed a significantly poorer RFS in the high-risk group compared with the low-risk group (HR = 12.69, 95% CI 3.55–45.34, *p* = 6.0E−07; Fig. 2A). After adjusting for gender, stage, CMS, MSI status, and age, multivariate Cox regression analysis demonstrated that 6-GPS was an independent predictor (HR = 20.21, 95% CI 4.16–98.12, *p* < 0.001; Fig. 2B). Notably, among all EOCRC samples in TCGA, 26 EOCRC samples were classified into the high-risk group, and 46 samples were classified into low-risk group based on 6-GPS. They were significantly different in terms of RFS (HR = 8.92, 95% CI 3.22–24.70, *p* < 0.0001; Additional file 1: Fig. S2); thus, we used these samples for follow-up analysis.

We applied Fisher’s exact test to evaluate the correlation between EOCRC subgroups and clinicopathological features. MSI status (*p* = 0.047; Fig. 2C) and CMS subtype (*p* = 0.014; Fig. 2D) showed statistically significant associations with EOCRC subgroups. High-frequency MSI (MSI-H) was found in a higher proportion of samples in high-risk EOCRC compared with in low-risk EOCRC (23.1% in high-risk EOCRC vs. 6.3% in low-risk EOCRC). We also found a higher proportion of CMS4 (50.0% in high-risk EOCRC vs. 20.8% in low-risk EOCRC) in high-risk EOCRC, but more CMS2 (54.2% in low-risk EOCRC vs. 19.2% in high-risk EOCRC) existed in low-risk EOCRC. It is known that CMS4 predicts worse RFS, while CMS2 predicts a better prognosis [36], which may partly explain the high recurrence rate in high-risk EOCRC.

### Verifying the performance of the 6-GPS in independent datasets

We applied it in two independent datasets to validate the performance of 6-GPS. For 28 surgery-only EOCRC patients in GSE39582, 3 patients were categorized as high-risk by 6-GPS with a marginally significant worse RFS compared to 25 low-risk patients (HR = 4.76, 95% CI 0.72–31.31, *p* = 0.086; Fig. 2E). Similar results were observed in 7 surgery-only EOCRC patients from GSE14333 (*p* = 0.090; log-rank test; Fig. 2F). Due to the small sample size of EOCRC, we combined GSE39582 and GSE14333, and surgery-only EOCRC patients in the combined dataset (*n* = 35) were significantly stratified in RFS (HR = 6.39, 95% CI 1.35–30.37, *p* = 9.80E−03; Fig. 2G).

We applied 6-GPS to classify EOCRC patients who received ACT in GSE39582, GSE14333, and combined dataset and uncover the role of the prognostic signature in treatment. Notably, in high-risk group, we found that patients who received ACT showed better RFS or DFS than those who did not; however, the difference was not statistically significant (HR = 0.34, 95% CI 0.05–2.43, *p* = 0.387 in GSE39582; HR = 0.36, 95% CI 0.03–4.36, *p* = 0.590 in GSE14333; HR = 0.34, 95% CI 0.08–1.53, *p* = 0.213 in combined dataset; Fig. 2E–G). In low-risk group, patients who received ACT showed significantly poorer RFS or DFS than those who did not (HR = 3.53, 95% CI 0.97–12.87, *p* = 0.043 in GSE39582; *p* = 0.190 in GSE14333; HR = 3.90, 95% CI 1.12–13.52, *p* = 0.015 in combined dataset; Fig. 2E–G). Furthermore, in GSE72970 and GSE104645, high-risk group showed a satisfactory response to ACT and had a therapeutic advantage over the low-risk group (Fig. 2H). Therefore, these findings suggest that patients with high-risk EOCRC may benefit from ACT.

### High-risk EOCRC showed high TMB

**Fig. 3:**
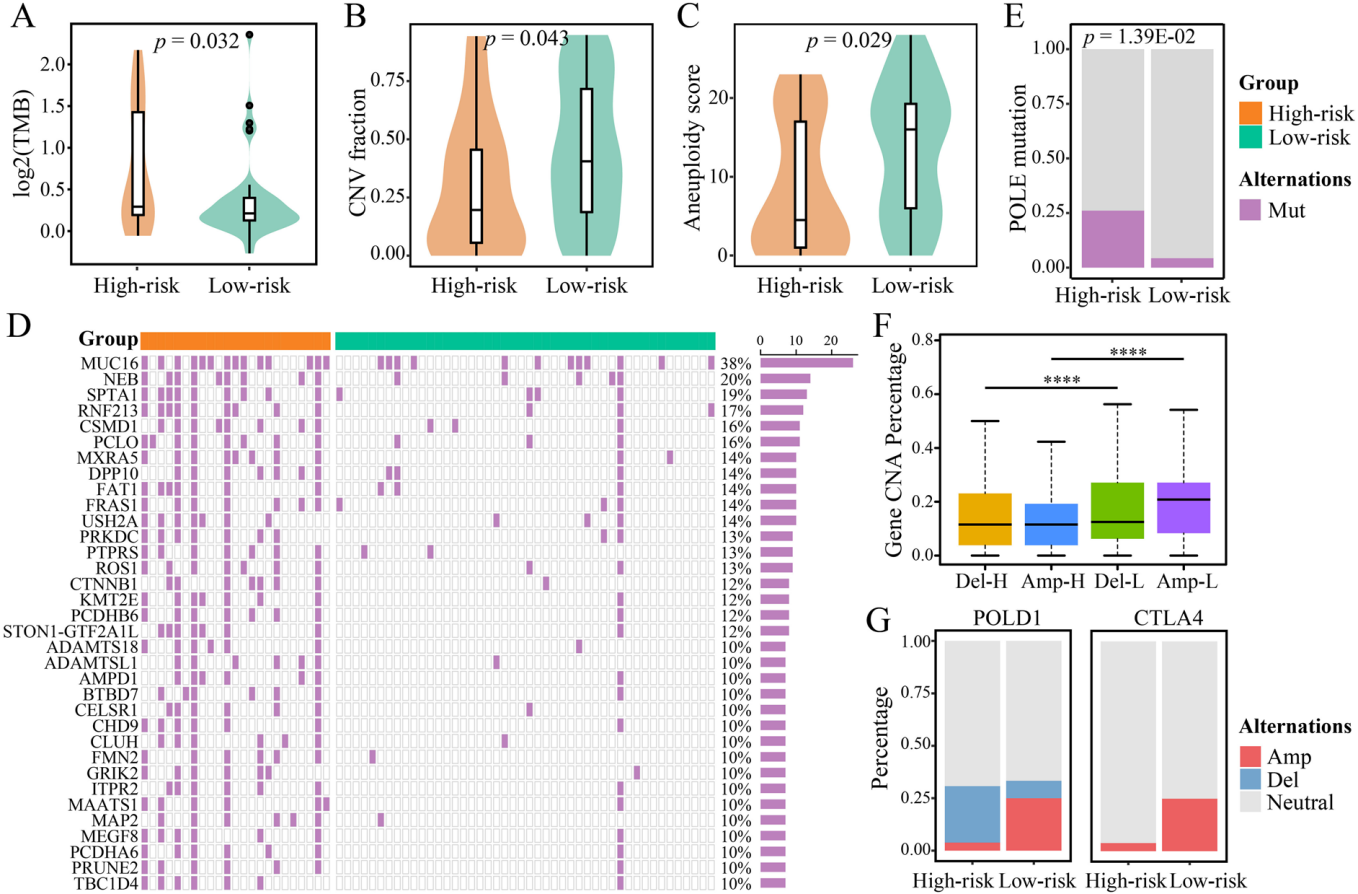
Genomic characteristics of high-risk and low-risk EOCRC samples. Differences in TMB (**A**), CNA fraction (**B**), and aneuploidy score (**C**) between high-risk and low-risk samples. **D** Heatmap depicting differentially mutated genes with mutation frequencies not less than 10% in the TCGA. **E** Difference in the frequency of POLE mutation between high-risk and low-risk samples. **F** Difference in the amplification and deletion of CNA between high-risk and low-risk samples. Amp-L, amplification in low-risk samples; Del-H, deletion in high-risk samples; Del-L, deletion in low-risk samples; ****p < 0.0001. **G** Histograms depicting the proportion of different CNA statuses of *CTLA4* and *POLD1* in high-risk and low-risk samples. Amp, amplification; Del, deletion; neutral, no change

In terms of global genomic alterations, high-risk samples displayed significantly higher TMB (*p* = 0.032; Wilcoxon rank-sum test; Fig. 3A), while low-risk samples displayed significantly higher CNA fraction (*p* = 0.043; Wilcoxon rank-sum test; Fig. 3B) and aneuploidy score (*p* = 0.029; Wilcoxon rank-sum test; Fig. 3C). High TMB may reflect better efficacy of immunotherapy in high-risk EOCRC.

It is known that higher TMB implies the presence of more mutant genes. Among the 69 EOCRC samples with mutation data, we discovered 184 genes with significantly high-frequency mutations in 23 high-risk samples compared to 46 low-risk samples (*p* < 0.05; Fisher’s exact test). The heatmap in Fig. 3D depicts differential mutation genes with mutation frequencies of at least 10%. Notably, among 184 differentially mutant genes, CRC driver genes *CTNNB1* (*p* = 1.41E−03), *MUC16* (*p* = 8.00E−03), and the gene encoding DNA polymerase epsilon (*POLE*; *p* = 1.39E-02; Fig. 3E) had significantly higher frequency of mutation in high-risk EOCRC.

As reported previously, *POLE* mutation may lead to additional mutations, which can increase TMB and is a source of cancer hypermutation. In addition, defects in mismatch repair genes, particularly at microsatellite sites, can lead to hypermutation of tumors and form the MSI-H phenotype. MSI-H tumors are a subset of high TMB tumors [37]. After excluding samples with MSI-H and *POLE* mutation, we found no difference in TMB between high-risk and low-risk EOCRC (*p* = 0.660; Wilcoxon rank-sum test; Additional file 1: Fig. S3A). Therefore, consistent with previous reports, high TMB in high-risk EOCRC is explained by MSI-H or *POLE* mutation. High-risk EOCRC showed poorer RFS than low-risk EOCRC after excluding hypermutation samples (*p* < 0.0001; Additional file 1: Fig. S3B). The results showed that the categorization ability of 6-GPS was retained in non-hypermutation EOCRC.

In addition to the somatic mutation profile, we compared CNA spectrums between the groups. For 74 EOCRC samples with CNA data, the results indicated that the frequency of amplification and deletion in 48 low-risk samples was significantly higher than that in 26 high-risk samples (*p* < 0.0001; Wilcoxon rank-sum test; Fig. 3F). We found that four genome regions, comprising one amplification region (20q) and three deletion regions (1p, 14q, and 18q), had significantly higher CNA in low-risk EOCRC (*p* < 0.05; Fisher’s exact test; Additional file 1: Fig. S3C). Notably, among genes highly associated with CRC, *CTLA4* and the gene encoding DNA polymerase delta (*POLD1*) had a higher frequency of amplification in low-risk EOCRC, while a higher frequency of *POLD1* deletion was observed in high-risk EOCRC (*p* < 0.05; Fisher’s exact test; Fig. 3G). These results suggest that there are distinct genomic differences between high-risk and low-risk EOCRCs.

### High-risk EOCRC showed highly invasiveness

**Fig. 4:**
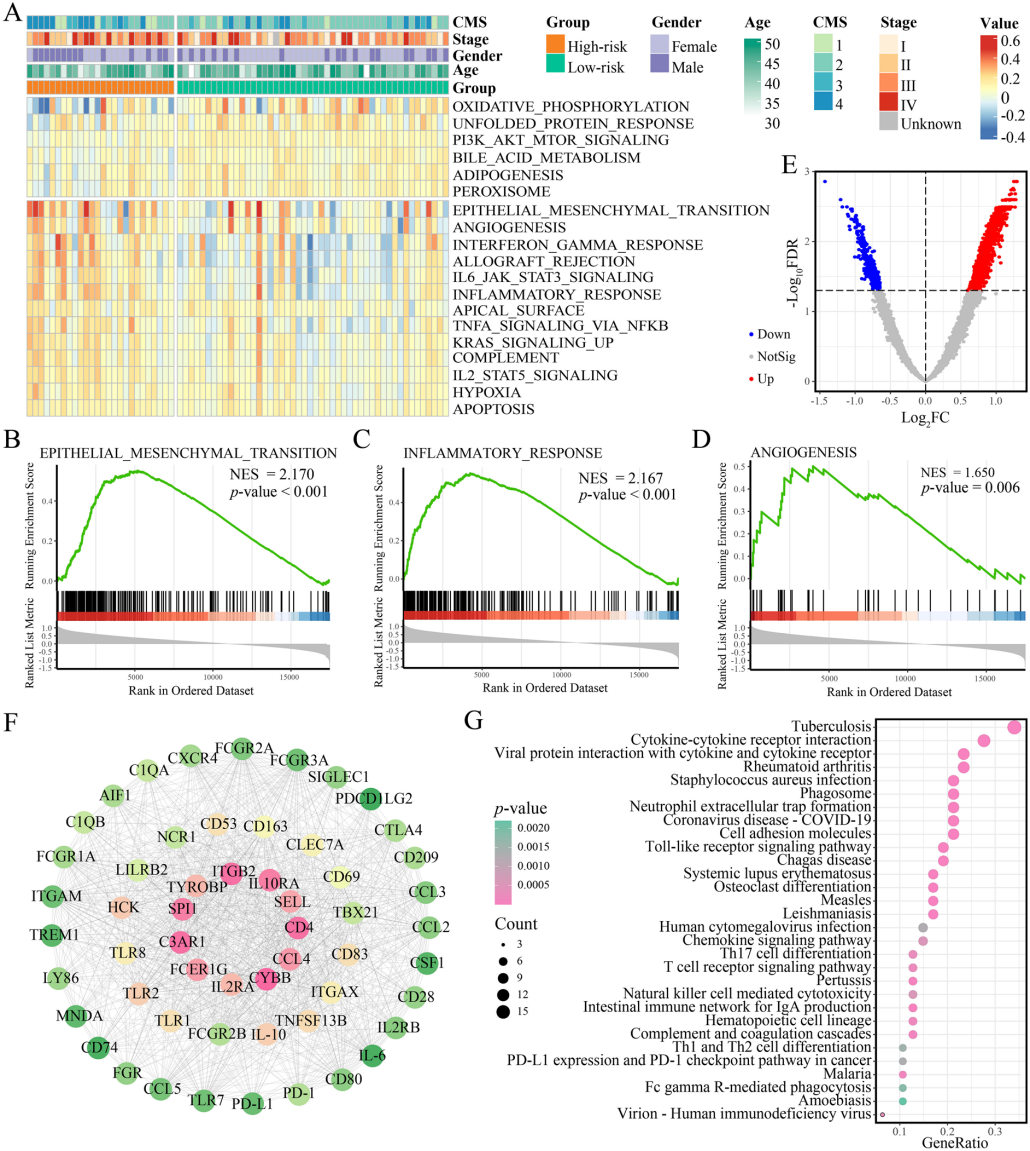
Pathway enrichment analysis between high-risk and low-risk EOCRC samples. **A** Heatmap displays the ssGSEA enrichment scores of hallmarks for high-risk and low-risk samples. GSEA shows EMT (**B**), inflammatory response (**C**), and angiogenesis (**D**) were upregulated in the high-risk group. **E** Volcano plot depicts DEGs between high-risk and low-risk samples. **F** PPI network of 55 DEGs. **G** The dot plot shows the top 30 significantly enriched pathways for KEGG

We characterized the molecular pathways specific for high-risk and low-risk EOCRCs via ssGSEA. For hallmarks, the enrichment scores of the epithelial-mesenchymal transition (EMT), KRAS signaling up, and angiogenesis were significantly higher in high-risk EOCRC, while low-risk EOCRC was enriched in bile acid metabolism (Fig. 4A). The GSEA analysis of hallmark gene sets were significantly enriched in the terms of the EMT, inflammatory response, and angiogenesis (Fig. 4B–D).

Differential analysis identified 2251 DEGs between high-risk and low-risk EOCRCs in TCGA, including 1894 upregulated genes and 357 downregulated genes (FDR < 0.05; limma; Fig. 4E). Subsequently, we screened the top 1000 DEGs for PPI network analysis. Finally, a topological network was constructed consisting of 55 immune-related genes, such as immune checkpoint genes (*PD-1*, *PD-L1*, and *CTLA4*), chemokines (*CCL2*, *CCL3*, *CXCR4*, and *CCL5*), and cytokines (*IL-10*, *CSF1*, and *TNFSF13B*). All of these genes were significantly upregulated in high-risk EOCRC (Fig. 4F). KEGG enrichment analysis revealed that 55 DEGs were significantly enriched in immune-related pathways like “Cytokine-cytokine receptor interaction”, “Chemokine signaling pathway”, and “T-cell receptor signaling pathway” (Fig. 4G).

Collectively, the regulatory pathways enriched in high-risk EOCRC tumors may contribute to higher invasiveness and recurrence.

### High-risk EOCRC showed high immune infiltration

**Fig. 5:**
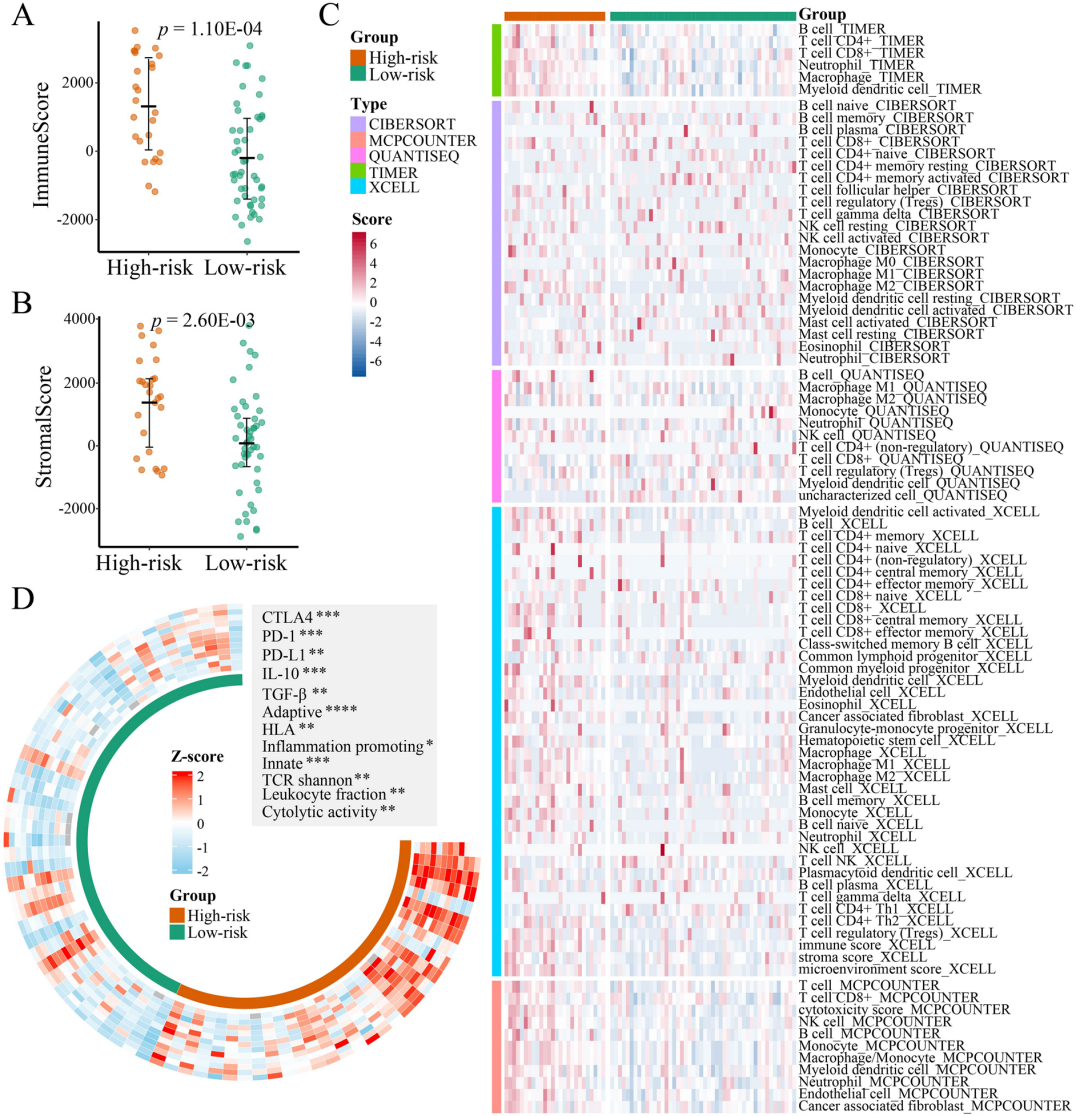
The differences in immune landscape between high-risk and low-risk EOCRC samples. ImmuneScore (**A**) and StromalScore (**B**) were compared between high-risk and low-risk samples. **C** Heatmap displaying differences in immune infiltration between high-risk and low-risk samples by CIBERSORT, MCP-counter, quanTIseq, TIMER, and xCell. **D** Circular heatmap depicting differences in immune signature scores and immune checkpoints between high-risk and low-risk samples. *p < 0.05; **p < 0.01; ***p < 0.001; ****p < 0.0001

The results of above enrichment analysis suggest that there may be differences in the tumor immune microenvironment between high-risk and low-risk EOCRCs, which necessitates more studies. Using the ESTIMATE method, we observed that immune score (*p* = 1.10E−04; Wilcoxon rank-sum test; Fig. 5A) and stromal score (*p* = 2.60E−03; Wilcoxon rank-sum test; Fig. 5B) were significantly higher in high-risk EOCRC than in low-risk EOCRC. Then, we investigated the difference in immune cell infiltration between high-risk and low-risk EOCRC using five transcriptome-based evaluation algorithms (TIMER, CIBERSORT, xCell, quanTIseq, and MCP-counter). The results demonstrated that high-risk EOCRC displayed higher infiltration of immune cells, such as macrophages, myeloid dendritic cells, and cancer-associated fibroblasts (Fig. 5C). CIBERSORT, quanTIseq, and xCell showed that compared with low-risk EOCRC, high-risk EOCRC showed higher infiltration of M2-like macrophages (*p* < 0.05; Wilcoxon rank-sum test; Fig. 5C). M2-like macrophages possess anti-inflammatory effects and promote tumor immune evasion, angiogenesis, growth, and metastasis and are associated with poor survival [38]. We found that anti-inflammatory cytokines, *IL-10* and *TGF-β*, produced by M2-like macrophages were significantly overexpressed in high-risk EOCRC and led to immunosuppression (*p* < 0.01; Wilcoxon rank-sum test; Fig. 5D).

Furthermore, we observed significantly higher TCR Shannon (*p* < 0.01; Wilcoxon rank-sum test; Fig. 5D) in high-risk EOCRC, indicating a stronger immune response. High-risk EOCRC also showed higher cytolytic activity, leukocyte fraction, adaptive and innate immune cells, HLA, and inflammation promoting scores (*p* < 0.05; Wilcoxon rank-sum test; Fig. 5D), which suggest that high-risk EOCRC has stronger antigen presentation ability. We also found that important immune checkpoint genes, such as *PD-1*, *PD-L1*, and *CTLA4*, were significantly upregulated in high-risk EOCRC (*p* < 0.05; Wilcoxon rank-sum test; Figs. 4F and 5D). These results suggest that high-risk EOCRC may be more sensitive to immune checkpoint inhibitors (ICIs).

### *SERPINE1*,* PECAM1*, *CDH1*, and *ANXA1* as recurrence-associated genes for EOCRC

**Fig. 6:**
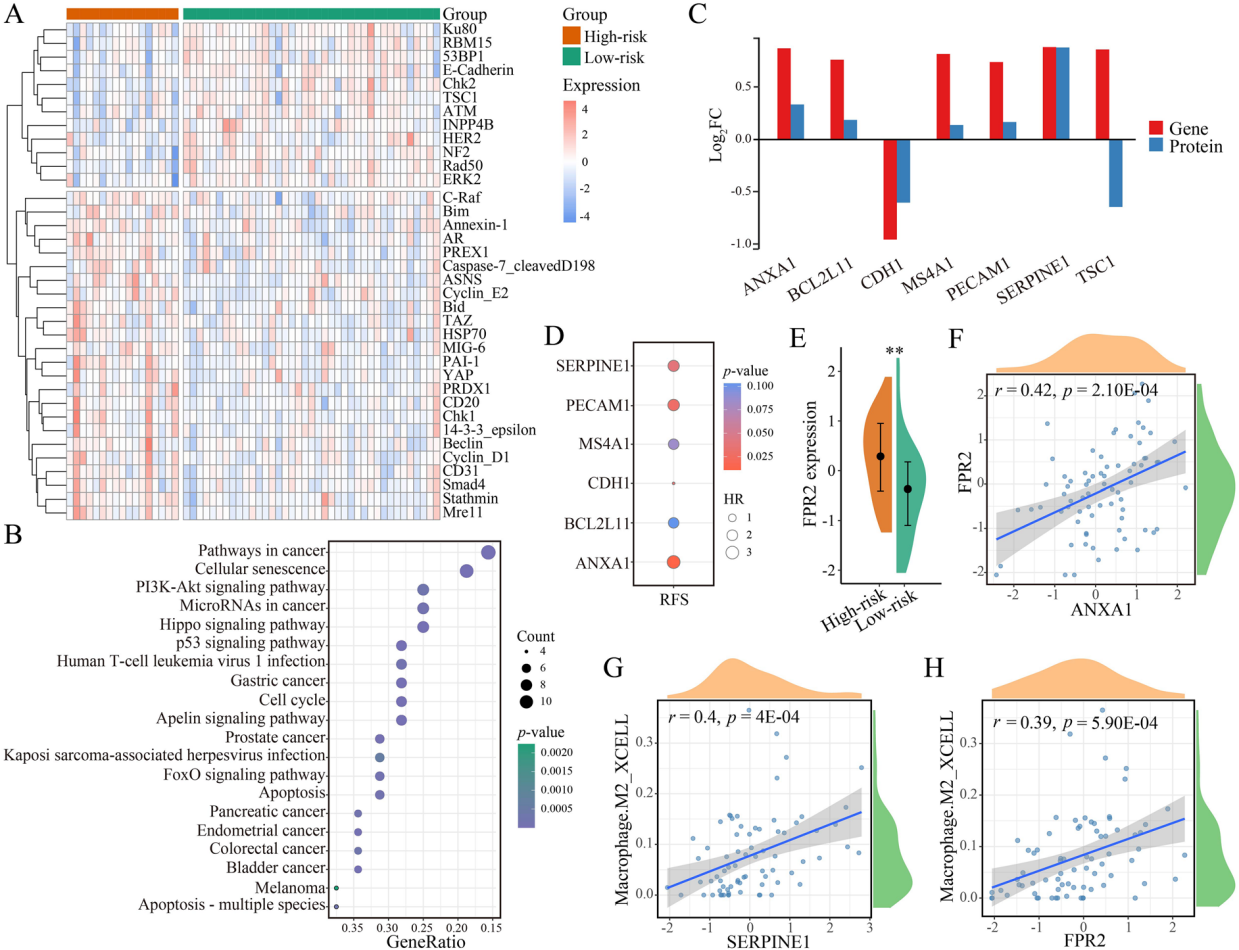
Identification of four recurrence-associated genes for EOCRC. **A** Heatmap depicting the expression of differential proteins in high-risk and low-risk groups. **B** KEGG pathway enrichment analysis of differential proteins. **C** Histogram showing dysregulation of the intersection of differential proteins and DEGs. **D** Correlation between 6 genes and RFS. **E** Differential expression of *FPR2* gene. **p < 0.01. **F** Correlation between the expression of *ANXA1* and *FPR2*. **G**, **H** Correlations between the expressions of genes (*SERPINE1* and *FPR2*) and M2-like macrophage infiltration

We analyzed the proteomes of high-risk and low-risk EOCRCs to observe downstream differences. For the 56 EOCRC samples with protein expression data, we found 51 proteins with significantly differential expression between 17 high-risk and 39 low-risk samples (*p* < 0.05; limma; Fig. 6A). For example, DNA damage repair proteins 53BP1 and TAM were upregulated in low-risk EOCRC, while YAP and Smad4 were upregulated in high-risk EOCRC.

We profiled the differentially expressed proteins via KEGG enrichment analysis and found that inflammatory pathways (“PI3K-AKT signaling pathway” and “p53 signaling pathway”) were significantly enriched (*p* < 0.05; Fig. 6B). For 7 differential proteins, seven genes that encode these proteins were also differentially expressed, and six of these genes showed the same dysregulation as their corresponding proteins (Fig. 6C). Of them, four genes showed prognostic significance (*p* < 0.05; univariate Cox regression); specifically, high expression of *SERPINE1*, *PECAM1*, and *ANXA1* was associated with poorer prognosis and short RFS, whereas high expression of *CDH1* was associated with a good prognosis, defining them as recurrence-associated genes for EOCRC (*p* < 0.05; Fig. 6D and Additional file 1: Fig. S4A–D).

*CD31*, encoded by *PECAM1*, is a tumor angiogenesis marker [39]. E-cadherin encoded by CDH1 is an important epithelial cell adhesion protein and a tumor suppressor. It is also an EMT-related marker [40]. Transcriptional downregulation of *CDH1* reduces cell adhesion and promotes CRC progression. These results substantiated the enrichment of angiogenesis and EMT in high-risk EOCRC. Protein plasminogen activator inhibitor-1 (PAI-1)-encoding gene, *SERPINE1*, is an oncogene involved in cell proliferation, cell survival, and regulation of tumor microenvironment (TME), which is associated with recurrence in CRC [41, 42]. Annexin 1, encoded by *ANXA1*, is an anti-inflammatory protein that modulates innate immune cell response as a downstream of glucocorticoids [43]. *FPR2* is the primary receptor responsible for the anti-inflammatory effects of *ANXA1*. *FPR2* was significantly upregulated in high-risk EOCRC (*p* < 0.01; Wilcoxon rank-sum test; Fig. 6E), and its expression was significantly and positively correlated with the expression of *ANXA1* (*r* = 0.42; *p* = 2.10E−04; Pearson’s correlation; Fig. 6F). In addition, *ANXA1* can enhance macrophage polarization toward an M2-like phenotype through *FPR2* and promote the immune suppression. High levels of *ANXA1* have been found in CRC, breast cancer, and melanoma, correlating with poor prognosis, low disease-free survival, and low overall survival [43].

In particular, we found that there was a significant and strongly positive correlation between the expression of *SERPINE1* and M2-like macrophage abundance in EOCRC (*r* = 0.4; *p* = 4E−04; Pearson’s correlation; Fig. 6G), which is consistent with previous studies reporting that *SERPINE1* expression promotes M2-like polarization of macrophages [38, 44]. Consistent with previous reports, the expression of *FPR2* showed a significant and strongly positive correlation with the abundance of M2-like macrophages (*r* = 0.39; *p* = 5.90E−04; Pearson’s correlation; Fig. 6H). These findings indicate that *SERPINE1*, *PECAM1*, *CDH1*, and *ANXA1* may be potential therapeutic targets for EOCRC.

## Discussion

Contrary to the declining trend in CRC-related mortality among people older than 50 years, the morbidity and mortality of EOCRC have been steadily rising. Thus, alterations in treatment strategies are needed to improve the survival of patients with EOCRC. Classifying EOCRC can help its treatment. Here, we developed a transcriptional signature based on 6-GPS to predict the recurrence risk of EOCRC and explore the mechanisms behind recurrence. The high-risk group exhibited higher invasiveness and TMB. Furthermore, there were significant differences in the tumor immune microenvironment between the high-risk and low-risk groups. Specifically, high-risk EOCRC displayed higher immune cell infiltration and immune scores and *PD-1*/*PD-L1* overexpression. High-TMB can predict response to ICI regardless of tumor type [45]. It has also been demonstrated that MSI-H and *POLE* mutation cause high TMB and may be potential biomarkers for predicting response to immunotherapy [46, 47]. Collectively, high-risk EOCRC showed a therapeutic preference for immunotherapy.

In this study, we identified four recurrence-associated genes, *SERPINE1*, *PECAM1*, *CDH1*, and *ANXA1*, whose expression affected the prognosis of EOCRC. They may be new therapeutic targets for EOCRC. Additionally, *SERPINE1* and *ANXA1* manipulated TME. *SERPINE1* overexpression may induce the high infiltration rate of M2-like macrophages in high-risk samples. *ANXA1* can promote macrophages polarization towards an M2-like phenotype via its receptor *FPR2*, thereby regulating the immune response. Therefore, inhibition of *ANXA1*/*FPR2* signaling may be an essential and creative immunotherapy strategy.

Tumor-associated macrophages (TAMs) are the most plastic and abundant immune cells in the TME. To date, all approved ICIs are monoclonal antibodies blocking *CTLA4*, *PD-1*, or *PD-L1*, with limited efficacy in most cases. The efficacy of immunotherapy is affected by several mechanisms, and TAMs play key roles in these mechanisms. Currently, there are four main strategies for macrophage-based therapy, including reducing macrophage recruitment, depletion of existing macrophages in the TME, repolarization of existing macrophages in the TME, and macrophage cell therapy [48]. Therefore, we believe that combining immune checkpoint inhibitors with targeted macrophage therapy may be an effective treatment option for patients with EOCRC who have a high risk of recurrence.

6-GPS is an REO-based signature which is robust against experimental batch effects and partial RNA degradation. 6-GPS is available and repeatable for clinical practice. It is important to acknowledge that our study has some potential limitations. Due to the insufficient EOCRC samples, we used EOCRC and LOCRC samples for developing the signature rather than EOCRC-only samples to identify prognosis-related gene pairs. However, we used 6-GPS in the EOCRC samples from TCGA to classify the EOCRC samples in the combined validation set into two groups with significantly different RFS. Therefore, we identified two groups with distinct recurrence risk and molecular profiles despite the relatively small sample size. We believe that future studies with adequate sample sizes can more evidently illuminate the importance of these findings.

## Conclusions

In this study, we developed and validated 6-GPS for predicting the recurrence risk of EOCRC for each patient. We explored the differences in the molecular mechanisms of EOCRC with distinct recurrence risks, which can provide valuable insights for developing new strategies for treating EOCRC. Our findings suggested that *SERPINE1*, *PECAM1*, *CDH1*, and *ANXA1* may be potential therapeutic targets for EOCRC with a high risk of recurrence. Further validation is needed in future large-sized studies.

### Supplementary Information

**Additional file 1: Table S1.** Clinicopathologic characteristics of EOCRC and LOCRC in TCGA. **Table S2.** The detailed information of 6-GPS. **Figure S1.** The survival differences between EOCRC and LOCRC. Kaplan–Meier curves depicting the RFS difference between EOCRC and LOCRC in TCGA (A) and GSE39582 (B). Kaplan–Meier curves depicting the DFS difference between EOCRC and LOCRC in GSE17538 (C) and GSE14333 (D). Kaplan–Meier curves depicting the OS difference between EOCRC and LOCRC in TCGA (E), GSE39582 (F), and GSE17538 (G). **Figure S2.** Kaplan–Meier curve depicting the RFS difference between high-risk and low-risk groups for all EOCRC patients in TCGA. **Figure S3.** Genomic analysis between high-risk and low-risk samples in TCGA. The effects of hypermutated tumors on the TMB (A) and 6-GPS classification ability (B). (C) Difference in the amplification and deletion of genomic regions between high-risk and low-risk samples. Amp, amplification; Del, deletion; neutral, no change. **Figure S4.** The association between *SERPINE1*, *CDH1*, *ANXA1*, and *PECAM1* expression and prognosis. (A-D) Kaplan–Meier curves depicting the survival difference between high and low expression of genes (*SERPINE1*, *CDH1*, *ANXA1*, and *PECAM1*) in EOCRC from TCGA.

## Data Availability

The datasets analyzed during the current study are available in the public database: Gene Expression Omnibus (GEO, https://www.ncbi.nlm.nih.gov/geo/) and cBioportal (https://www.cbioportal.org/).
