# Supplementary material for: Identifying individualized prognostic signature and unraveling the molecular mechanism of recurrence in early-onset colorectal cancer
Source: Eur J Med Res. 2023 Nov 20;28:533. doi: 10.1186/s40001-023-01491-y (PMC10658991; doi:10.1186/s40001-023-01491-y)
Supplement: Supplementary file 1 — Additional file 1: Table S1. Clinicopathologic characteristics of EOCRC and LOCRC in TCGA. Table S2. The detailed information of 6-GPS. Figure S1. The survival differences between EOCRC and LOCRC. Kaplan–Meier curves depicting the RFS difference between EOCRC and LOCRC in TCGA (A) and GSE39582 (B). Kaplan–Meier curves depicting the DFS difference between EOCRC and LOCRC in GSE17538 (C) and GSE14333 (D). Kaplan–Meier curves depicting the OS difference between EOCRC and LOCRC in TCGA (E), GSE39582 (F), and GSE17538 (G). Figure S2. Kaplan–Meier curve depicting the RFS difference between high-risk and low-risk groups for all EOCRC patients in TCGA. Figure S3. Genomic analysis between high-risk and low-risk samples in TCGA. The effects of hypermutated tumors on the TMB (A) and 6-GPS classification ability (B). (C) Difference in the amplification and deletion of genomic regions between high-risk and low-risk samples. Amp, amplification; Del, deletion; neutral, no change. Figure S4. The association between SERPINE1, CDH1, ANXA1, and PECAM1 expression and prognosis. (A-D) Kaplan–Meier curves depicting the survival difference between high and low expression of genes (SERPINE1, CDH1, ANXA1, and PECAM1) in EOCRC from TCGA. [file 40001_2023_1491_MOESM1_ESM.docx]

**Table S1** Clinicopathologic characteristics of EOCRC and LOCRC in TCGA.

| **Variables** | **EOCRC (n = 74)** | **LOCRC (n = 401)** | ***p*-value** |
| --- | --- | --- | --- |
| **Gender** |  |  |  |
| Male | 32 (43.2%) | 220 (54.9%) | 0.075 |
| Female | 42 (56.8%) | 181 (45.1%) |  |
| **Stage** |  |  |  |
| I | 9 (12.2%) | 64 (16.0%) | 0.024 |
| II | 19 (25.7%) | 162 (40.4%) |  |
| III | 29 (39.2%) | 107 (26.7%) |  |
| IV | 16 (21.6%) | 51 (12.7%) |  |
| Unknown | 1 (1.4%) | 7 (1.8%) |  |
| **CMS** |  |  |  |
| 1 | 9 (12.2%) | 73 (18.2%) | 0.602 |
| 2 | 31 (41.9%) | 156 (38.9%) |  |
| 3 | 11 (14.9%) | 64 (16.0%) |  |
| 4 | 23 (31.1%) | 108 (26.9%) |  |
| **MSI** **status** |  |  |  |
| MSS | 42 (56.8%) | 252 (62.8%) | 0.456 |
| MSI-L | 17 (23.0%) | 65 (16.2%) |  |
| MSI-H | 9 (12.2%) | 58 (14.5%) |  |
| Unknown | 6 (8.1%) | 26 (6.5%) |  |
| **Neoadjuvant/Radiation**  **Therapy** |  |  |  |
| Yes | 8 (10.8%) | 15 (3.70%) | 0.016 |
| No | 66 (89.2%) | 386 (96.3%) |  |

MSI, microsatellite instability; MSS, microsatellite stable; MSI-L, low-frequency microsatellite instability; MSI-H, high-frequency microsatellite instability.

**Table S2** The detailed information of 6-GPS.

| ***G_i_*** | ***G_j_*** | ***p*-value** | **C-index** |
| --- | --- | --- | --- |
| *HPS6* | *DKK1* | 7.09E-03 | 0.582 |
| *NR2E3* | *CCDC40* | 7.37E-03 | 0.576 |
| *WNT5B* | *TNNI2* | 1.21E-02 | 0.565 |
| *TP53TG1* | *B4GALT3* | 3.97E-02 | 0.535 |
| *RGS12* | *HPCAL4* | 1.22E-02 | 0.557 |
| *MAPK8IP1* | *KCNRG* | 6.20E-03 | 0.569 |

C-index, concordance index.

**
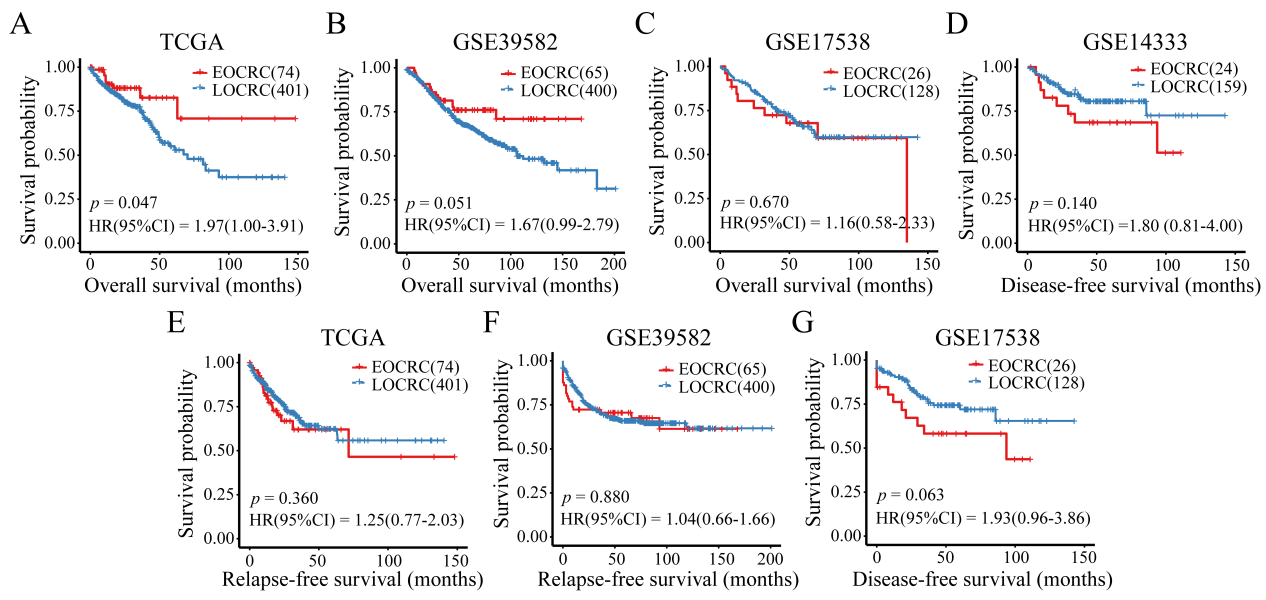
**

**Figure S1** The survival differences between EOCRC and LOCRC. Kaplan-Meier curves depicting the RFS difference between EOCRC and LOCRC in TCGA (**A**) and GSE39582 (**B**). Kaplan-Meier curves depicting the DFS difference between EOCRC and LOCRC in GSE17538 (**C**) and GSE14333 (**D**). Kaplan-Meier curves depicting the OS difference between EOCRC and LOCRC in TCGA (**E**), GSE39582 (**F**), and GSE17538 (**G**).

**
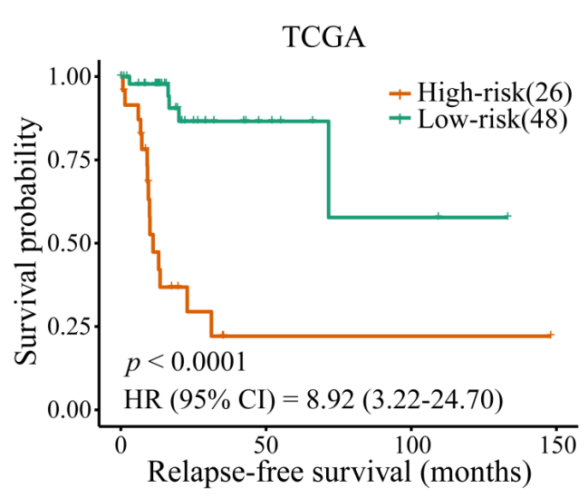
**

**Figure S2** Kaplan-Meier curve depicting the RFS difference between high-risk and low-risk groups for all EOCRC patients in TCGA.

**
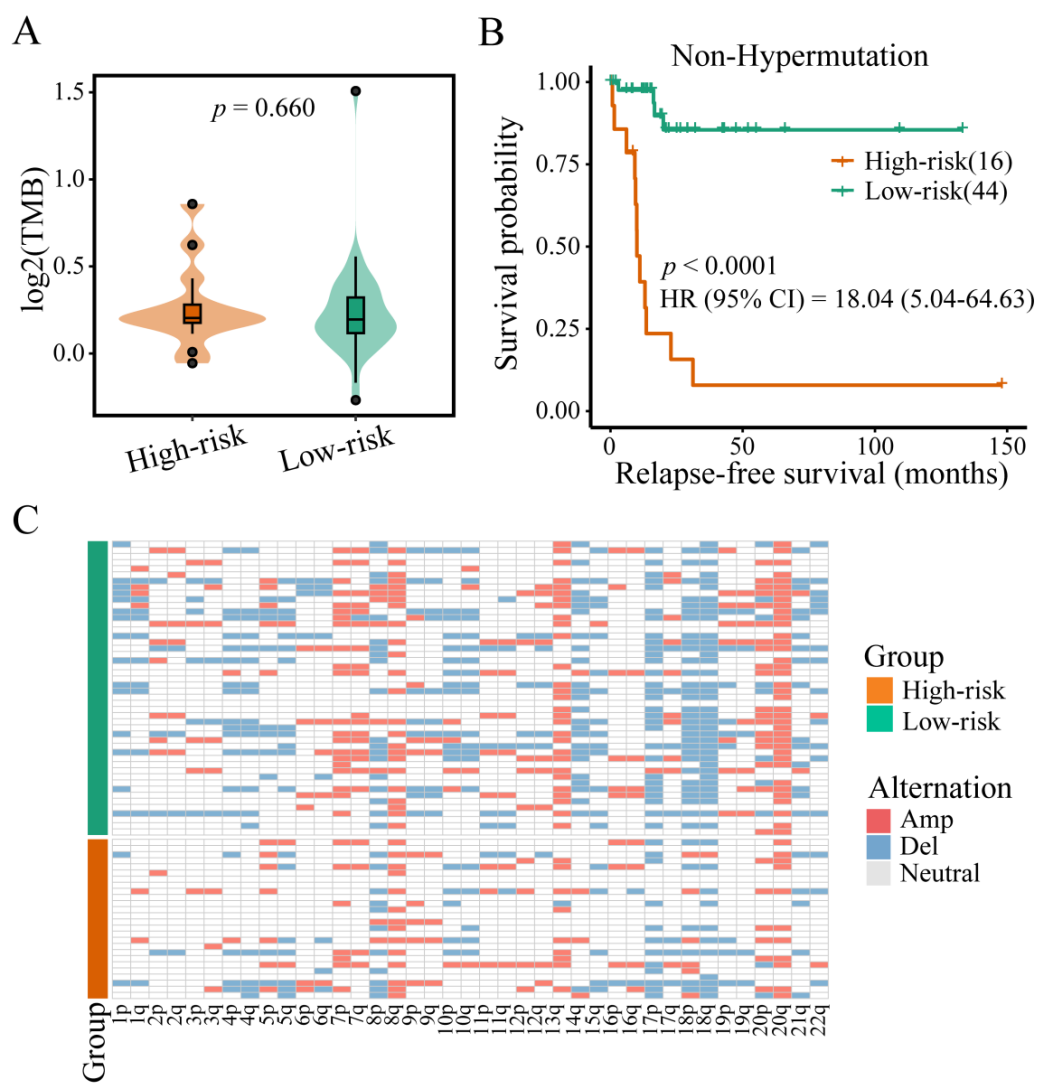
**

**Figure S3** Genomic analysis between high-risk and low-risk samples in TCGA. The effects of hypermutated tumors on the TMB (**A**) and 6-GPS classification ability (**B**). (**C)** Difference in the amplification and deletion of genomic regions between high-risk and low-risk samples. Amp, amplification; Del, deletion; neutral, no change.


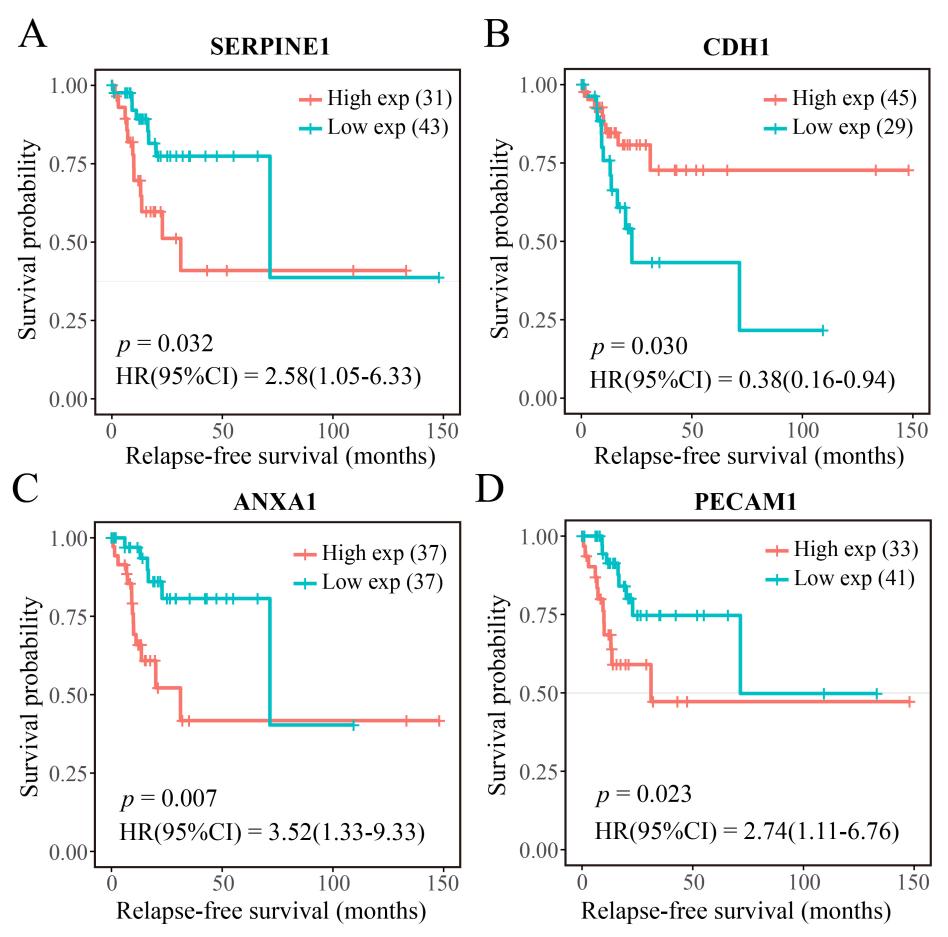


**Figure S4** The association between *SERPINE1*, *CDH1*, *ANXA1*, and *PECAM1* expression and prognosis. (**A-D**) Kaplan-Meier curves depicting the survival difference between high and low expression of genes (*SERPINE1*, *CDH1*, *ANXA1*, and *PECAM1*) in EOCRC from TCGA.
